# Geographical patterning of sixteen goat breeds from Italy, Albania and Greece assessed by Single Nucleotide Polymorphisms

**DOI:** 10.1186/1472-6785-9-20

**Published:** 2009-09-02

**Authors:** Lorraine Pariset, Antonella Cuteri, Christina Ligda, Paolo Ajmone-Marsan, Alessio Valentini, ECONOGENE Consortium

**Affiliations:** 1 Dipartimento di Produzioni Animali, Università degli Studi della Tuscia, Viterbo, Italy; 2 National Agricultural Research Foundation, Thessaloniki, Greece; 3 Istituto di Zootecnica, Università Cattolica del Sacro Cuore, Piacenza, Italy; 4

## Abstract

**Background:**

SNP data of goats of three Mediterranean countries were used for population studies and reconstruction of geographical patterning. 496 individuals belonging to Italian, Albanian and Greek breeds were genotyped to assess the basic population parameters.

**Results:**

A total of 26 SNPs were used, for a total of 12,896 genotypes assayed. Statistical analysis revealed that breeds are not so similar in terms of genetic variability, as reported in studies performed using different markers. The Mantel test showed a strongly significant correlation between genetic and geographic distance. Also, PCA analysis revealed that breeds are grouped according to geographical origin, with the exception of the Greek Skopelos breed.

**Conclusion:**

Our data point out that the use of SNP markers to analyze a wider breed sample could help in understanding the recent evolutionary history of domestic goats. We found correlation between genetic diversity and geographic distance. Also PCA analysis shows that the breeds are well differentiated, with good correspondence to geographical locations, thus confirming the correlation between geographical and genetic distances. This suggests that migration history of the species played a pivotal role in the present-day structure of the breeds and a scenario in which coastal routes were easier for migrating in comparison with inland routes. A westward coastal route to Italy through Greece could have led to gene flow along the Northern Mediterranean.

## Background

The domestic goat (*Capra hircus*) has been an important livestock resource since its domestication [1-3].

Even now, goats are of great importance in many developing countries, to exploit marginal agricultural resources, and in developed countries, for the production of high quality products and the achievement of sustainable development of rural areas. However, breeding programs and selection schemes in goats are less advanced than in other livestock.

Archaeological evidence indicates a probable migration of the Neolithic farmers out of the Near East and across Europe following two main routes, through the continental heartland up the Danube valley or along the Mediterranean coast [4-7] crossing the sea to the major islands. Archaeological data and radiocarbon dates on seeds or bones provide support for an earlier arrival in western Europe via the Mediterranean route rather than the "Danubian" route [8]. In addition, genetic data revealed a weaker degree of phylogeographic structuring in domestic goats compared to other livestock species [9,10], which probably results from high gene flow at the intercontinental level, suggesting that goats have been extensively transported [2,11]. This led to initial settlement in the Balkans and southern Italy [12]. A decrease of genetic diversity likely occurred during this colonization process in Europe [13].

The Mediterranean Sea had a key role in the history of livestock also in post-Neolithic times, when civilisations like Phoenicians, Greeks, Romans and Berbers probably introduced new species of animals and new breeds of livestock in southwest Europe arriving by sea. Some colonists may have improved local livestock importing stock from overseas [14,15], explaining the unexpectedly high diversity in breeds of domestic goats [16], the differential cattle migration along the Mediterranean coast [14] and the close genetic relationship between Tuscan cattle breeds and Near Eastern breeds [17]. The role of the Mediterranean Sea as a natural corridor connecting the peninsula to the Near East, North Africa, and southern Europe is particularly plausible for domestic sheep and goat, species adaptable to various environments and easy to transport [15].

A relative lack of breed standardization, herdbook breeding, parentage control and rigorous management may have facilitated gene flow between geographically nearby breeds in south-eastern Europe [18]. Gene flow may also have a historical background as goats were actively traded all over the Mediterranean basin during the Phoenician, Greek and Roman periods. However, in some circumstances, gene flow is limited by distance and local management, which reduces the effective population size as a result of genetic isolation.

So far, studies on genetic diversity of goats have focused on Swiss [19] and Asian breeds [20-26]. Only a few were relative to Mediterranean breeds [27-29], but all of these employed mitochondrial markers. A wide-range analysis of goat diversity in Europe and the Middle East has been conducted using microsatellites [18]. The use of microsatellites, the most common method used today to estimate neutral genetic diversity [30,31], presents disadvantages such as null alleles, interpretation difficulty of allele calling and size homoplasy [32]. Single nucleotide polymorphisms (SNPs) are useful markers for estimating parameters such as population history and inference of relationships [33,34] and they could potentially become the marker of choice in ecological and conservation studies [35,36]. The use of these markers is promoted also by a rapid development of genotyping techniques [32,37,38].

We applied SNP genotyping to the study of goat breeds from Italy, Albania and Greece to analyse genetic diversity of goats in these areas.

## Results

### Population Genetics

A total of 26 SNPs identified as polymorphic on a panel of European goat breeds [39] were used to genotype 496 individuals belonging to 6 Albanian, 2 Greek and 8 Italian breeds, for a total of 12,896 genotypes assayed. Loci were analysed to identify SNPs under selection in a separated study [40]. Analysis were then performed excluding the three SNPs in two loci identified as outliers, even if the use of all the 26 SNPs did not lead to substantial differences in the results.

Expected heterozygosity of the loci ranged from 0.016 (FABP4) to 0.495 (GHR), with a mean of 0.300. *F**IS* per population ranged from 0.017 (Liquenasi) to 0.197 (Capore) (Table 1). Observed heterozygosity of the loci determined from SNP frequencies (Table 2) ranged from 0.012 (FABP4) to 0.463 (GHR), with a mean of 0.272.

**Table 1:** Breeds analysed, country of origin and their sample sizes (N), average observed heterozygosity (*Ho*), average expected heterozygosity (*He*) and *F**IS* calculated per locus per sample and averaged over populations.

**Breed**	**label**	**Origin**	**N**	**Ho**	**He**	***F**IS***
Argentata dell' Etna^2^	ARG	Italy	31	0.280	0.322	0.131
Bionda dell' Adamello^2^	BIO	Italy	31	0.318	0.340	0.066
Camosciata	CAM	Italy	31	0.273	0.330	0.173
Capore	CAP	Albania	31	0.221	0.275	0.197
Dukati	DUK	Albania	31	0.295	0.309	0.047
Girgentana^1,2^	GIR	Italy	32	0.274	0.286	0.041
Greek goat	GRG	Greece	31	0.293	0.327	0.104
Grigia molisana^1,2^	GMO	Italy	31	0.292	0.306	0.048
Hasi	HAS	Albania	31	0.272	0.307	0.115
Liquenasi	LIQ	Albania	31	0.296	0.301	0.017
Mati	MAT	Albania	30	0.246	0.273	0.100
Muzhake	MUZ	Albania	31	0.259	0.295	0.124
Orobica^2^	ORO	Italy	31	0.287	0.295	0.030
Sarda	SAR	Italy	31	0.287	0.311	0.078
Skopelos2	SKO	Greece	31	0.177	0.201	0.122
Valdostana^1,2^	VAL	Italy	31	0.293	0.308	0.047
Mean				0.273	0.299	0.090

^1 ^Listed by FAO as endangered or critical . ^2 ^Number of breeding females below the EU thresholds (<10000). (Commission Regulation (EC), n. 445/2002).

**Table 2:** *Locus*, SNP, major allele frequency (m.a.f), observed heterozygosity (*Ho*), expected heterozygosity (*He*), *Fst *values and deviations from Hardy-Weinberg equilibrium (p-value) for each genotyped SNP.

***locus***	**SNP**	**m.a.f.**	**Ho**	**He**	***F**ST***	**p-value**
Activin receptor IIB	*ACVR2B*	0.803	0.308	0.294	0.076	0.815
Calpastatin	*CAST*	0.948	0.096	0.087	0.124	0.742
K-casein	*CSN3*	0.761	0.314	0.348	0.046	0.660
CathepsinK	*CTSK*	0.945	0.098	0.102	0.029	0.997
Desmin	*DES*	0.602	0.452	0.457	0.051	0.999
MHC class II DQA gene	*HLA-DQA_1*	0.884	0.116	0.191	0.077	0.000*
MHC class II DQA gene	*HLA-DQA_2*	0.672	0.158	0.400	0.102	0.000*
MHC class II DRB gene	*HLA-DRB*	0.838	0.226	0.258	0.057	0.077
Fatty acid-binding protein 4	*FABP4*	0.992	0.012	0.016	0.009	0.096
Fibronectin	*FN1*	0.811	0.238	0.292	0.050	0.309
Growth differentiation factor 9B	*GDF9*	0.808	0.215	0.298	0.043	0.004*
Growth hormone receptor	*GHR*	0.514	0.463	0.495	0.011	0.794
Interleukin-4	*IL4*	0.508	0.440	0.432	0.144	0.932
Interleukin-2	*IL2_1*	0.983	0.033	0.032	0.008	1.000
Interleukin-2	*IL2_2*	0.839	0.243	0.246	0.096	0.964
Integrin B1	*ITGB1*	0.637	0.422	0.452	0.025	0.947
Beta-lactoglobulin	*LGB*	0.757	0.338	0.341	0.078	0.998
Melatonin	*MTNR1A*	0.558	0.453	0.451	0.093	0.865
Myostatin	*GDF8*	0.942	0.098	0.106	0.031	0.940
Prion protein	*PRNP_1*	0.624	0.422	0.439	0.070	0.965
Prion protein	*PRNP_2*	0.649	0.408	0.431	0.058	0.641
Toll-like receptor 4	*TLR4*	0.571	0.441	0.475	0.033	0.231
microsatellite	*U80*	0.838	0.254	0.262	0.037	0.996
	*Mean*	0.706	0.272	0.300	0.059	

*: Highly significant

The frequencies of the major alleles are reported in table 2 and ranged from 0.508 for the locus IL4 to 0.992 for the locus FABP4. Except for IL2-1 and FABP4, showing frequencies of the rare alleles of 0.017 and 0.008, respectively, all other SNPs have a frequency of the rare allele greater than 5%, as observed for the same loci on a different European breeds panel [39]. Also mean observed and expected heterozygosity showed similar values to those reported in the same paper. The frequencies of the major alleles per SNP and per population are presented in Additional file 1. Some populations presented fixed alleles in a number of SNPs. Particularly, Skopelos and Girgentana showed the highest number of fixed alleles (5). SNPs FABP4 and IL2_1 are fixed in 12 and 8 populations, respectively (Additional file 1).

Significant deviations from Hardy-Weinberg equilibrium over all populations (p-value < 0.005) were observed in three loci (Table 2). Significant deviations from Hardy-Weinberg equilibrium (HWE) for each locus and population were observed for five loci. Locus CSN3 was not in HWE in Muzake population. Locus HLA-DQA_1 was not in HWE in Hasi, Skopelos and Argentata dell'Etna populations. HLA-DQA_ 2 was not in HWE in seven populations, five of which are Italian: Capore, Argentata dell'Etna, Bionda dell'Adamello, Camosciata, Sarda, Valdostana. Locus HLA-DRB is not in HWE in two populations: Capore and Greek Goat. Locus TLR4 was not in HWE in Camosciata population only.

Significant deviations from linkage equilibrium (p-value < 0.01) were observed for two of the analysed locus pairs: HLA-DQA_2 and GDF9 loci, PRP_1 and PRP_2. Loci have not yet been mapped in the goat; HLA-DQA_2 and GDF9 are located on *Bos taurus *chromosome 23 and 7, respectively; the two SNPs on PRP are 458 bp apart on *B. taurus *chromosome 13 . The log-likelihood based test of genotypic differentiation for all populations showed statistically significant values (p-value < 0.01) except for GHR, IL2_1 and FABP4 loci which have very little informativeness.

The analysis revealed an overall *F**ST* of 0.063. That is, 6.3% of allelic variation was accounted across breeds and 93.7% within breeds. Weir and Cockerham's [41] estimate of *F**ST* per locus ranged from 0.008 (IL2_1) to 0.144 (IL4), with a mean of 0.059 (Table 2). Pairwise *F**ST* values are given in Table 3. *F**ST* estimates, between Skopelos and Valdostana (0.19), Orobica (0.19), Camosciata (0.22), Girgentana (0.18) e Argentata dell'Etna (0.15) populations, were higher than all other pairwise comparisons suggesting the Skopelos as the most diverse population.

**Table 3:** Pairwise *F**ST* below diagonal, Nei's standard distance (Nei 1972) above diagonal.

	**ARG**	**BIO**	**CAM**	**CAP**	**DUK**	**GIR**	**GMO**	**GRG**	**HAS**	**LIQ**	**MAT**	**MUZ**	**ORO**	**SAR**	**SKO**	**VAL**
**ARG**	0	0.033	0.080	0.036	0.075	0.063	0.045	0.038	0.043	0.035	0.061	0.037	0.103	0.018	0.167	0.085
**BIO**	0.013	0	0.067	0.065	0.078	0.063	0.067	0.045	0.062	0.058	0.067	0.050	0.083	0.029	0.156	0.056
**CAM**	0.058	0.044	0	0.132	0.127	0.094	0.115	0.068	0.097	0.090	0.136	0.116	0.138	0.050	0.231	0.114
**CAP**	0.016	0.043	0.110	0	0.068	0.064	0.052	0.027	0.032	0.033	0.036	0.023	0.116	0.045	0.106	0.107
**DUK**	0.056	0.059	0.108	0.048	0	0.118	0.061	0.068	0.053	0.039	0.036	0.035	0.129	0.077	0.114	0.127
**GIR**	0.045	0.044	0.074	0.044	0.102	0	0.106	0.056	0.098	0.088	0.097	0.093	0.156	0.046	0.193	0.104
**GMO**	0.026	0.047	0.094	0.032	0.042	0.089	0	0.051	0.060	0.027	0.071	0.054	0.127	0.053	0.106	0.117
**GRG**	0.019	0.025	0.046	0.006	0.050	0.037	0.032	0	0.031	0.038	0.048	0.037	0.093	0.027	0.116	0.074
**HAS**	0.024	0.042	0.077	0.011	0.034	0.081	0.041	0.011	0	0.038	0.036	0.028	0.091	0.051	0.139	0.106
**LIQ**	0.016	0.038	0.068	0.012	0.020	0.071	0.009	0.019	0.018	0	0.041	0.020	0.096	0.037	0.101	0.110
**MAT**	0.043	0.047	0.117	0.016	0.017	0.080	0.053	0.029	0.017	0.022	0	0.017	0.099	0.067	0.090	0.105
**MUZ**	0.018	0.029	0.096	0.002	0.015	0.075	0.035	0.018	0.008	0.001	-0.003	0	0.094	0.045	0.090	0.084
**ORO**	0.086	0.064	0.120	0.097	0.112	0.140	0.111	0.075	0.073	0.079	0.081	0.076	0	0.114	0.204	0.145
**SAR**	-0.002	0.007	0.027	0.023	0.058	0.027	0.034	0.007	0.031	0.018	0.047	0.025	0.096	0	0.139	0.086
**SKO**	0.150	0.142	0.221	0.088	0.098	0.180	0.090	0.100	0.123	0.085	0.073	0.072	0.189	0.121	0	0.199
**VAL**	0.065	0.034	0.091	0.086	0.110	0.086	0.099	0.054	0.086	0.091	0.087	0.065	0.128	0.065	0.187	0

The Mantel test showed a strongly significant correlation between genetic and geographic distances (0.40, p-value < 0.001, over 1000 permutations).

### PCA analysis

**Figure 1:**
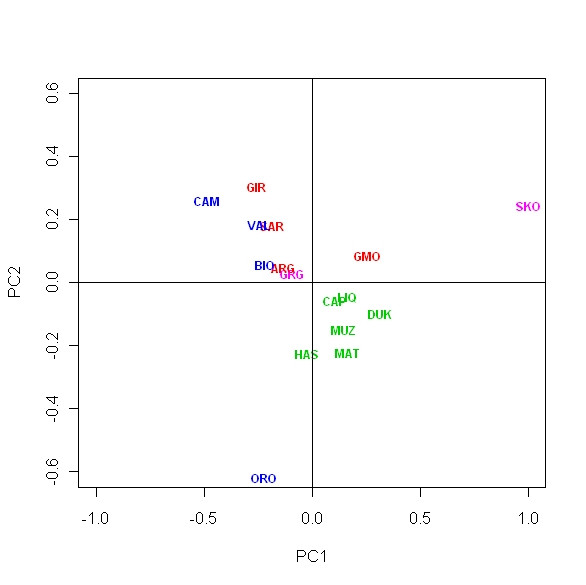
**Principal component analysis (PCA) of allele frequencies from twenty three SNP loci genotyped in the sixteen goat breeds**. Projection on axis 1 and axis 2, which cumulatively explained 52% of the total inertia contained in the data set. Breeds acronyms as in Table 1. Albanian breeds, green; North Italian breeds, blue; South Italian breeds, red; Greek breeds, pink.

Genetic relationships were also explored by means of principal component analysis. The coefficients of the linear combinations reveal which SNPs most affect the component value. As for the first component, SNP IL4 presents extreme positive and SNP LGB extreme negative values, respectively. Likewise, the second component is mostly affected by the SNPs ACVR2B and MTNR1A, with positive sign, and by the SNPs HLA-DQA_2, IL4 and LGB with negative sign. To examine the overall pattern of population differentiation, PCA was conducted with the first two axes, which cumulatively explained 52% of the total inertia contained in the data set (Figure 1). Breeds are grouped according to geographical origin, with the exception of the Greek Skopelos breed.

### Genetic distance

Distance-based phylogenetic analysis was used to describe the relationships between breeds. Table 3 presents the Nei [42] genetic distance relating the 16 goat breeds studied. The lowest distance values are observed between Muzhake and Mati (0.017) and Sarda and Argentata dell'Etna (0.018). The Greek Skopelos breed results the most distant one, very distant from all the 4 breeds of northern Italy and from Argentata dell'Etna and Girgentana.

## Discussion

Archaeological evidence showed that two main colonization routes took place in Europe after the initial domestication events in the Fertile Crescent: the Mediterranean route and the Danubian route. Cañon et al. [18], using microsatellites, report a decrease in genetic diversity as well as an increase in the level of differentiation at the breed level from south-east to north-west in European goat breeds, supporting the hypothesis of migration of domestic livestock from the Middle East towards western and northern Europe.

Our results indicate that a highly significant correlation between genetic and geographic distance exists. The presence of a geographic component in genetic diversity was already reported in breeds of Northern and Southern Italy in a previous study using SNPs [43] and it is confirmed here in a larger area. Such a geographic component is generally not observed when using mitochondrial markers. As reported by Luikart et al. [11] only 10% of the variance assessed by mtDNA is partitioned among continents. This could be due to the nature of the markers used for the analysis, as suggested by Naderi et al. [44]. In fact, mtDNA informativeness is limited because it does not detect male-mediated gene flow and does not predict the nuclear genomic diversity [45]. In the paper by Naderi et al. [44] the breeds cannot be distinguished on the basis of mtDNA, even if authors report that more than 77% of the mtDNA variation is found within breeds, while there is a low regional differentiations of haplotypes. At a regional scale, the lack of geographic structure has been reported using mtDNA in different regions [16,25,28] with the exception of one paper [24].

From PCA analysis the breeds appear well differentiated with 52% of the variance explained by the first two principal components. There is also a good correspondence to geographical locations, thus confirming the correlation between geographical and genetic distances identified by the Mantel test. PCA indicates a westward route to southern Italy through Greece, that may suggest contacts between Albania and Italian peninsula and between Greece and Italian Islands (Sardinia and Sicily). In post-Neolithic times, some colonists may have improved local livestock as well as importing stock from overseas. The transport of animals made by sea has been already proposed for cattle [14,17] and goats [15,18]. The role of the Mediterranean Sea as a natural corridor connecting the Italian peninsula to the Near East, North Africa, and southern Europe is particularly plausible for small sized species, as sheep and goats species adaptable to various environments and easy to transport during human migration and commercial trade [4,11,45].

The Greek Skopelos breed results the most distant one, reflecting the fact that it has been raised only in a island and on the mainland of Magnisia. The distance is not due to inbreeding as *F**IS* = 0.122, not the highest value in our breeds (max *F**IS* = 0.197 in Capore), but to the lack of admixture with other populations since long time and possibly a natural selection versus local environment. The Skopelos breed is largely differentiated from the other goat populations in Greece, both morphologically and in terms of performance. According to the inhabitants of the Skopelos island, the goat used to live in an uninhabited small island of Northern Sporades, and it was recently domesticated. The breed is also said to have some relationship with the wild goat of the Gioura island, originated from the homonymous island [46]. Breeders, due to the favourable characteristics of the breed (high prolificacy and high milk production), established a breeders association and applied a genetic improvement programme since 1981. Also the Orobica is very far apart from the other breeds. Again the distance is not attributable to inbreeding (*F**IS* = 0.030), but to isolation of this breed in a very secluded valley of Italian Alps.

Among the Italian southern breeds, it is interesting that the lowest distance is seen between Argentata dell'Etna, from Sicily, and Sarda, original from Sardinia. The two islands, although quite far apart, were important trade posts of Phoenician, Punic and Roman traders.

**Figure 2:**
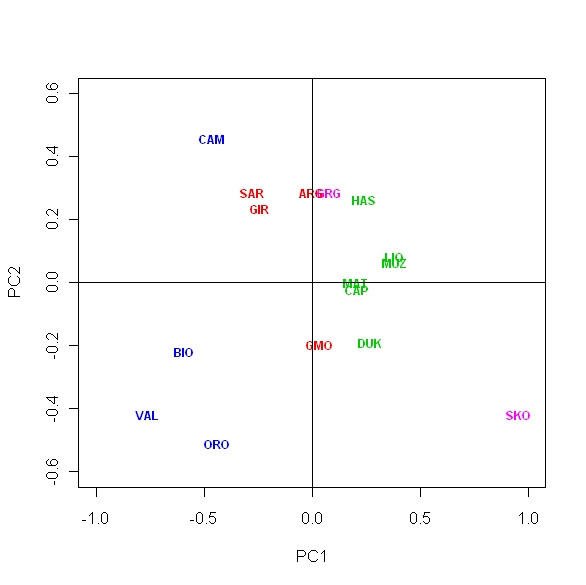
**Principal component analysis (PCA) of allele frequencies from twenty six SNP loci genotyped in the sixteen goat breeds**. Projection on axis 1 and axis 2, which cumulatively explained 56.8% of the total inertia contained in the data set. Breeds acronyms as in Table 1. Albanian breeds, green; North Italian breeds, blue; South Italian breeds, red; Greek breeds, pink.

The analysis carried so far excluded SNPs that were proven under selection [40]. If we include these SNPs (CSN1S1_1, CSN1S1_2 and LIPE, [39]) we find that the overall distance pattern remains unchanged but for two breeds of Northern Italy, Bionda and particularly Valdostana, that become closer to Orobica (Figure 2). This is due to casein and LIPE allele frequency that are almost fixed in these breeds for the same allele, while the average allele frequency for the other breeds is 50% (Additional file 1). Caseins have been the first genes to be associated to milk production, characteristics and curding properties [47-49]. It is noteworthy that milk production and cheese making is a primary agricultural activity in North Italy since historical times as demonstrated by the high frequency of lactase persistence in humans [50]. We hypothesize that converged selection for caseins and LIPE (an enzyme important for cheese making as well [51]) occurred in Orobica, Valdostana and Bionda dell'Adamello breeds making them "similar" for what concerns their exploitation objectives.

## Conclusion

Our data point out that the use of SNP markers to analyze a wider breed sample, until now scarcely employed for genetic population studies in livestock, could help in understanding the recent evolutionary history of domestic goats.

We found correlation between genetic diversity and geographic distance. Also PCA analysis shows that the breeds are well differentiated, with good correspondence to geographical locations, thus confirming the correlation between geographical and genetic distances. This suggests that migration history of the species played a pivotal role in the present-day structure of the breeds. Instead, the limited genetic similarity within main geographical areas suggests that breed differentiation could have occurred in more recent times, after the main migrations.

On the basis of the observed gradient of genetic diversity decreasing from south-east to north-west, and of the signals of the northward dispersal of populations from the domestication centre, we hypothesize that coastal routes from the domestication centre to Italy through Greece could be a likely explanation for the observed gene flow along the Northern Mediterranean.

## Methods

### Material

**Figure 3:**
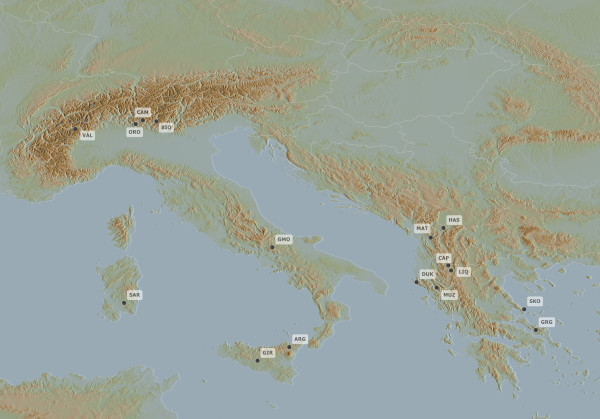
**Rearing area of each analysed breed**.

Blood samples of a total of 496 goats, about one third male, were collected in farms spread over the traditional rearing area of each breed (Figure 3). No more than 3 unrelated individuals per flock, from an average of 10 farms per breed, were sampled to reduce the relationship among animals and to increase the breed representativeness. Samples were obtained following the rules of each of the countries involved in sampling. Wherever possible, we used part of samples taken by public veterinaries within national animal health plans. A total of 16 breeds were analysed. The breed names, their acronyms, countries of origin, and the sample sizes are given in Table 1. DNA, extracted by phenol-clorophorm or commercial kits in the relative sampling laboratory, was tested for quality and concentration by electrophoresis on 0.8% agarose gel, stained with ethidium bromide and compared to a commercial standard.

### SNP analysis

SNPs characterization has been described elsewhere [39]. SNP ascertainment bias was minimised by sequencing target DNA in at least 8 individuals from different populations [39]. Large scale genotyping of all animals was performed by outsourcing to a commercial genotyping company . Generally, accuracy greater than 99% was achieved. Quality control criteria were adopted (water as negative control, inter plate duplicate testing of a known DNA, intra plate testing of a known DNA). All the SNPs described in [39] were genotyped on our samples. In this investigation 23 SNPs in 19 genes and in one microsatellite were used (Table 2), excluding SNPs showing a frequency >0.05 on our samples.

### Data analysis

Allele frequencies were calculated using *F**STAT* 2.93[52]. Observed and expected heterozygosities (*H**O* and *H**E*, respectively), Weir and Cockerham's [41] estimate of *F**IS* per population, of *F**ST* per locus and population pairs were calculated for each locus using *G**ENEPOP* 4.0 [53]. The same software was used to test deviations from Hardy-Weinberg equilibrium (HWE) for each locus and population and for locus over all populations using a Markov chain of 100 000 steps and 1000 dememorization steps and to assess deviations from genotypic linkage disequilibrium (LD) for each pair of loci using the same Markov chain parameters as for the HWE test. We performed the Probability-test (the "exact HW test").

Isolation by distance (IBD, [54]) has been assessed by plotting the genetic distance among population pairs as a function of the geographic distance between those pairs to check whether more distant population pairs are more different genetically, providing information on the genetic structure of the populations and [55]. We applied the Mantel test [56,57] implemented in the ade4 library of *R *2.6.0 open source software (publicly available at ) to estimate the correlation between pairwise *F**ST* values and pairwise geographic distance using 1000 replicates to test significance. The matrix of the geographic distance was computed using the coordinates obtained with a global positioning system (GPS).

A principal component analysis (PCA) was performed on the covariance matrix of SNP frequency data to investigate spatial patterns of genetic variation using the *R *2.6.0 open source software.

Nei [42] genetic distances between populations pairs were calculated to obtain relative estimates of the time that has passed since the populations were established using *POWERMARKER*[58].

## Authors' contributions

LP: designed the study, carried out SNP data analysis and drafted the manuscript. AC: completed most of the statistical analysis. CL: provided Greek samples and formulated parts of the paper. PAM: leader of the Econogene project, provided DNA samples and guidance for the project. AV: participated in developing ideas, in supervision and revision of the manuscript. All authors read and approved the final manuscript.

## Supplementary Material

Additional file 1 **Table S1**. Frequency of the Major allele per SNP and per population. Click here for file
