# Supplementary material for: Geographical patterning of sixteen goat breeds from Italy, Albania and Greece assessed by Single Nucleotide Polymorphisms
Source: BMC Ecol. 2009 Sep 2;9:20. doi: 10.1186/1472-6785-9-20 (PMC2754418; doi:10.1186/1472-6785-9-20)
Supplement: Additional file 1 — Table S1. Frequency of the Major allele per SNP and per population. [file 1472-6785-9-20-S1.doc]

# Additional file 1*.* Frequency of the Major allele per SNP and per population.

| **Marker** | CAP | DUK | HAS | LIQ | MAT | MUZ | GRG | SKO | ARG | BIO | CAM | GIR | GMO | ORO | SAR | VAL | All |
| --- | --- | --- | --- | --- | --- | --- | --- | --- | --- | --- | --- | --- | --- | --- | --- | --- | --- |
| **ACVR2B** | 0.83 | 0.70 | 0.73 | 0.85 | 0.81 | 0.74 | 0.72 | 0.87 | 0.90 | 0.87 | 0.88 | 0.97 | 0.92 | 0.50 | 0.91 | 0.68 | 0.80 |
| **CAST** | 0.98 | 0.98 | 0.98 | 1.00 | 0.98 | 1.00 | 0.97 | 0.98 | 0.97 | 0.72 | 0.95 | 1.00 | 0.97 | 0.76 | 0.98 | 0.93 | 0.95 |
| **CSN3** | 0.93 | 0.69 | 0.74 | 0.84 | 0.75 | 0.82 | 0.81 | 0.73 | 0.92 | 0.68 | 0.60 | 0.75 | 0.79 | 0.84 | 0.79 | 0.52 | 0.76 |
| **CTSK** | 0.98 | 0.94 | 0.91 | 1.00 | 0.98 | 0.97 | 0.96 | 0.95 | 1.00 | 0.90 | 0.88 | 0.88 | 0.95 | 1.00 | 0.97 | 0.85 | 0.95 |
| **DES** | 0.71 | 0.35 | 0.59 | 0.62 | 0.55 | 0.67 | 0.55 | 0.76 | 0.68 | 0.62 | 0.33 | 0.48 | 0.73 | 0.68 | 0.55 | 0.73 | 0.60 |
| **HLA-DQA_1** | 1.00 | 0.91 | 0.91 | 0.81 | 0.91 | 0.86 | 0.90 | 0.93 | 0.79 | 0.88 | 0.57 | 1.00 | 0.95 | 0.94 | 0.76 | 0.96 | 0.88 |
| **HLA-DQA_2** | 0.58 | 0.85 | 0.81 | 0.75 | 0.73 | 0.80 | 0.57 | 0.80 | 0.64 | 0.70 | 0.58 | 0.20 | 0.76 | 0.81 | 0.62 | 0.46 | 0.67 |
| **HLA-DRB** | 0.88 | 0.95 | 0.86 | 0.70 | 1.00 | 0.91 | 0.81 | 0.94 | 0.87 | 0.89 | 0.64 | 0.84 | 0.75 | 0.64 | 0.85 | 0.83 | 0.84 |
| **FABP4** | 1.00 | 1.00 | 1.00 | 0.98 | 1.00 | 1.00 | 1.00 | 1.00 | 1.00 | 0.95 | 1.00 | 1.00 | 1.00 | 1.00 | 0.98 | 0.96 | 0.99 |
| **FN1** | 0.80 | 0.86 | 0.66 | 0.91 | 0.80 | 0.89 | 0.64 | 1.00 | 0.75 | 0.87 | 0.77 | 0.90 | 0.66 | 0.89 | 0.86 | 0.72 | 0.81 |
| **GDF9** | 0.80 | 0.76 | 0.84 | 0.77 | 0.86 | 0.80 | 0.89 | 0.92 | 0.61 | 0.82 | 0.90 | 0.80 | 0.66 | 1.00 | 0.69 | 0.82 | 0.81 |
| **GHR** | 0.48 | 0.58 | 0.65 | 0.58 | 0.53 | 0.55 | 0.48 | 0.53 | 0.50 | 0.40 | 0.68 | 0.37 | 0.50 | 0.40 | 0.47 | 0.58 | 0.51 |
| **IL4** | 0.62 | 0.72 | 0.61 | 0.65 | 0.69 | 0.55 | 0.48 | 0.76 | 0.39 | 0.27 | 0.32 | 0.47 | 0.65 | 0.60 | 0.39 | 0.00 | 0.51 |
| **IL2_1** | 0.98 | 0.98 | 0.98 | 0.97 | 1.00 | 0.95 | 0.95 | 0.97 | 1.00 | 1.00 | 0.95 | 1.00 | 1.00 | 1.00 | 1.00 | 1.00 | 0.98 |
| **IL2_2** | 0.97 | 0.89 | 0.91 | 0.87 | 0.80 | 0.88 | 0.87 | 1.00 | 0.82 | 0.77 | 0.70 | 0.89 | 0.90 | 0.48 | 0.92 | 0.74 | 0.84 |
| **ITGB1** | 0.70 | 0.48 | 0.76 | 0.62 | 0.55 | 0.55 | 0.73 | 0.69 | 0.67 | 0.60 | 0.74 | 0.70 | 0.62 | 0.68 | 0.71 | 0.40 | 0.64 |
| **LGB** | 0.79 | 0.50 | 0.84 | 0.63 | 0.83 | 0.76 | 0.83 | NA | 0.72 | 0.77 | 0.83 | 0.83 | 0.48 | 0.95 | 0.84 | 0.78 | 0.76 |
| **MTNR1A** | 0.50 | 0.50 | 0.37 | 0.57 | 0.46 | 0.48 | 0.67 | 1.00 | 0.41 | 0.50 | 0.63 | 0.48 | 0.72 | 0.39 | 0.63 | 0.56 | 0.56 |
| **GDF8** | 0.84 | 0.93 | 0.83 | 0.97 | 0.98 | 0.93 | 0.90 | 0.98 | 0.90 | 0.98 | 1.00 | 1.00 | 0.96 | 0.95 | 0.94 | 0.97 | 0.94 |
| **PRNP_1** | 0.66 | 0.72 | 0.62 | 0.60 | 0.82 | 0.73 | 0.58 | 0.94 | 0.55 | 0.53 | 0.32 | 0.52 | 0.55 | 0.67 | 0.52 | 0.68 | 0.62 |
| **PRNP_2** | 0.70 | 0.68 | 0.63 | 0.66 | 0.82 | 0.72 | 0.61 | 0.95 | 0.57 | 0.57 | 0.36 | 0.53 | 0.67 | 0.66 | 0.50 | 0.72 | 0.65 |
| **TLR4** | 0.47 | 0.52 | 0.60 | 0.60 | 0.53 | 0.48 | 0.52 | 0.44 | 0.61 | 0.60 | 0.62 | 0.45 | 0.69 | 0.89 | 0.60 | 0.52 | 0.57 |
| **U80** | 0.95 | 0.85 | 0.94 | 0.95 | 0.90 | 0.92 | 0.89 | 0.86 | 0.79 | 0.72 | 0.73 | 0.72 | 0.84 | 0.73 | 0.77 | 0.89 | 0.84 |
